# Optimization of cotton variety registration criteria aided with a genotype-by-trait biplot analysis

**DOI:** 10.1038/s41598-017-17631-4

**Published:** 2017-12-08

**Authors:** Naiyin Xu, Michel Fok, Jian Li, Xiaoni Yang, Weikai Yan

**Affiliations:** 1 Institute of Industrial Crops, Jiangsu Academy of Agricultural Sciences/Key Laboratory of Cotton and Rapeseed, Ministry of Agriculture, Nanjing, 210014 China; 2 CIRAD, UPR AIDA, F-34398 Montpellier, France; 3 AIDA, Univ Montpellier, CIRAD, Montpellier, France; 4 Ottawa Research and Development Center, Agriculture & Agri-Food Canada, 960 Carling Ave., Neatby Building, Ottawa, ON K1A 0C6 Canada

## Abstract

China is one of the largest cotton producing countries in the world thanks to high yields, on which a variety registration system has mainly focused, so that a lack of quality is nowadays acknowledged as a weak point of the cotton industry in that country. The objective of this study was to check the hypothesis that bias in cultivar selection in favor of yield has been maintained through the application of an imperfect selection index (SI), but that a better outcome is possible. Our demonstration is based on an analysis of the data from ten years of cotton variety trials using genotype-by-trait biplots, implemented both for the cultivar selection index (SI) currently applied in China and for an adjusted selection index (ASI) that more effectively took into account the antagonism between yield and quality traits. The main findings were: 1) significant negative associations between yield and fiber quality hindered their simultaneous improvement; 2) registered genotypes were mainly determined by the SI which was primarily yield-oriented; 3) no progress in fiber quality was recorded unlike yield; 4) balanced progress in yield and quality is possible through an adjusted selection index (ASI) guided by genotype-by-trait biplot analysis.

## Introduction

China has been the main producer of upland cotton (*Gossypium hirsutum* L.) since 1984, and was only caught up recently by India, as the result of an active breeding program. Since the introduction of upland cotton to China from the United States in the 19th century, more than 1000 cotton varieties have been developed and utilized in the main cotton planting regions there^1^. Cotton yield per unit area has been increased by more than nine-fold between the cultivars released in the 1950’s to those released in 2015, leading China to rank among countries with the highest yields in the world^2^.

However, the pace of improvement in cotton fiber quality has lagged behind that of yield^3^ and the lack of fiber quality is acknowledged to be one of the main shortfalls of the cotton sector in China^4^ for meeting the requirements of the modern spinning industry. While the narrow genetic base and the lack of competitive marketing price for high-quality fiber have commonly been mentioned as responsible factors, the influence of the official criteria applied to register new cultivars has not been sufficiently assessed, if at all. The extent to which cotton yield in the field has been promoted at the expense of quality criteria has yet to be appraised.

Cotton fiber quality is defined by several physical properties (fiber length, fiber strength, elongation, reflectance, yellowness, length uniformity index, spinning consistency index and micronaire as an index of fiber maturity and fineness) and breeding for varieties with better quality is not simple. The various quality traits are often negatively correlated making it difficult, if not impossible, to create varieties that are simultaneously optimal for all traits.

Globally and historically, the selection criteria and weights for various target traits have varied with breeding programs, registration systems, countries, and sub-regions (mega-environments)^5^. In China, since the initiation of the variety registration system in 1990, candidate genotypes have been evaluated for their multiple traits and only expert opinions prevail in cultivar registration decisions. In 2007, the Chinese Ministry of Agriculture issued the *Standards for Registration of Cotton Varieties* ^6^, introducing a selection index combining various breeding objectives, such as fiber length, strength, micronaire, lint yield and pre-frost yield rate (as an indicator of earliness) to help register new varieties that give high yield and good quality fiber.

The objective of this paper was to check the hypothesis that the bias in favor of yield, consequently to the detriment of quality, has continued in spite of the application of a selection index in China, but a better outcome is possible. In our demonstration, we also show that a lapse of ten years is sufficient to reveal breeding bias. Our demonstration is based on an analysis of a GGE (genotype + genotype by environment interaction) biplot and more precisely a particular type called a genotype-by-trait (GT) biplot. The GGE-biplot has been rather intensely used to help genotype evaluation^7,8^, the selection of adequate and non-redundant locations in multi-location varietal trials, or to identify mega-environments among locations^9,10^ where variety trials are conducted. A GT-biplot is an alternative use of the GGE-biplot to clarify the trait profiles of genotypes^11^. In a GT-biplot, the term “environment” or “location” in the GGE biplot is replaced by “trait” but data must be scaled to be unit neutral^12^. The objectives of genotype analysis by traits are twofold. One is to understand the relations existing between traits, synergy or antagonism, particularly between those that are key breeding objectives. The other is to understand the trait profiles^13^ of the genotypes, as each genotype may be regarded as a package of traits. It is the package (trait profile), rather than a single trait, that determines the usefulness and superiority of a genotype.

In practice, this article addresses the following questions: Did the application of the selection index properly address the associations between? Could the outcome be improved through an adjusted selection index (ASI)? In this study, GT-biplot analyses were based on data from the cotton regional trials in the Yangtze River Valley (YRV) from 2006 to 2015.

## Results

### Associations between traits and trait profiles of genotypes

**Figure 1:**
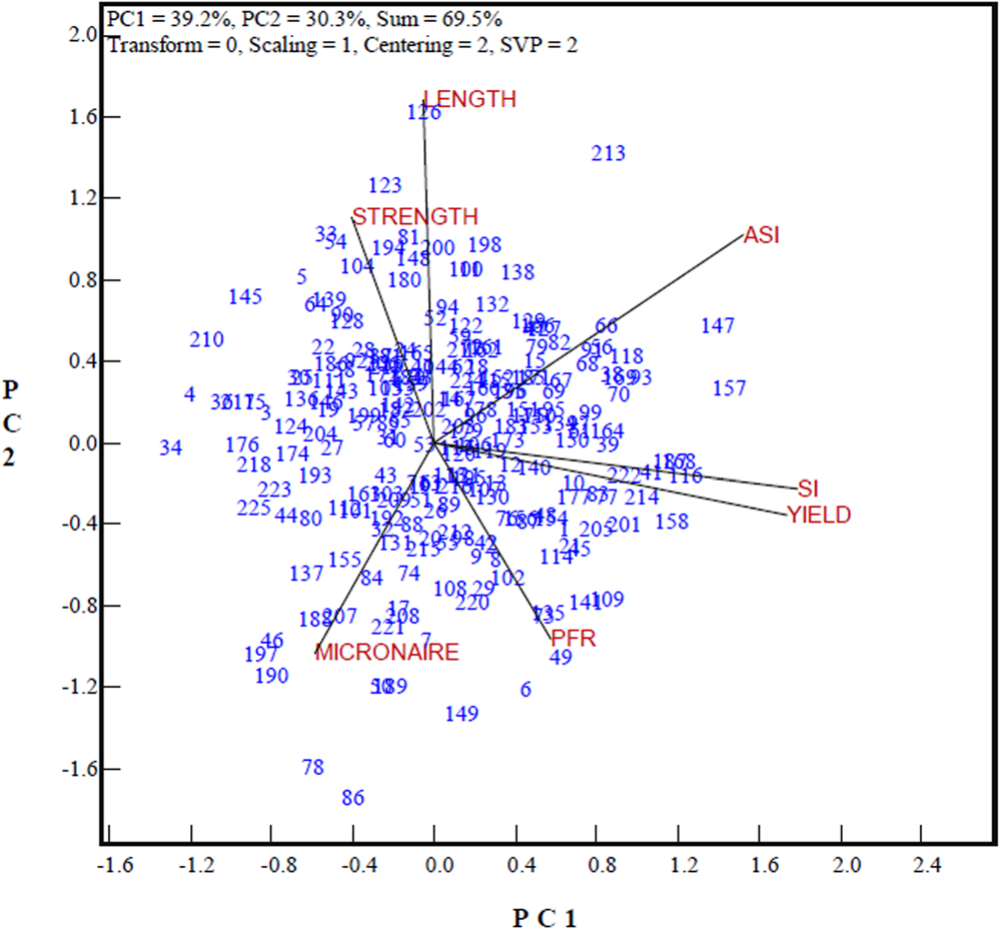
The genotype-by-trait (GT) biplot to show the trait associations across genotypes for five target traits plus two selection indices. The traits are fiber length, strength, micronaire, pre-frost yield rate (PFR), lint yield relative to check (YIELD), selection index (SI) and adjusted selection index (ASI). SVP, singular value partitioning method.

The GT-biplot (Fig. 1) displayed 69.5% of the information in the standardized data of the 225 genotypes for five traits plus the selection index (SI) and the adjusted selection index (ASI), whose components and weights are presented in Table 1. This biplot can be examined from three perspectives.

**Table 1:** Trait max-scaled data and selection indices for 225 cotton genotypes tested in the Yangtze River Valley from 2006 to 2015 (not all the genotypes are shown).

Code	Genotype^*^	Length	Strength	Micronaire	Yield	PFR	SI	ASI	Selected for SI	Selected for ASI	Culled for^**^
126	Qm8	1.00	0.76	0.08	0.23	0.51	0.33	0.77	No	Yes	Yield_L
148	Sh05	0.71	0.83	0.23	0.26	0.60	0.40	0.67	No	Yes	Yield_L
123	Nz0822	0.86	0.61	0.08	0.22	0.40	0.27	0.64	No	Yes	Yield_L
81	Hzmh321	0.81	0.46	0.15	0.37	0.29	0.35	0.62	No	Yes	Yield_L
158	Sy328	0.48	0.22	0.38	0.95	0.79	0.90	0.73	Yes	Yes	Strength_L
1	20_9f1	0.33	0.21	0.27	0.76	0.60	0.74	0.59	Yes	No	Strength_L
109	Km12	0.37	0.21	0.62	0.97	0.60	0.86	0.59	Yes	No	Strength_L
48	Ga18_Hb	0.49	0.21	0.46	0.79	0.56	0.70	0.57	Yes	No	Strength_L
141	Sch18	0.33	0.18	0.46	0.83	0.75	0.78	0.56	Yes	No	Strength_L
49	Ga19_Hb	0.21	0.12	0.46	0.81	0.73	0.77	0.49	Yes	No	Strength_L
6	B8130r	0.23	0.13	0.54	0.74	0.87	0.71	0.44	Yes	No	Strength_L
114	Lyht1	0.41	0.55	0.81	0.87	0.59	0.83	0.60	Yes	No	Micronaire_H
135	Rhm10	0.23	0.79	0.92	0.84	0.67	0.90	0.59	Yes	No	Micronaire_H
213	Z1104	0.93	0.67	0.00	0.57	0.70	0.66	0.96	Yes	Yes	
147	Sh01_3	0.59	0.41	0.00	0.88	0.64	0.92	0.93	Yes	Yes	
…	…	…	…	…	…	…	…	…	…	…	
200	Xz008f1	0.86	0.91	0.54	0.43	0.55	0.49	0.72	No	Yes	
201	Xz198	0.32	0.63	0.50	0.82	0.80	0.91	0.72	Yes	Yes	
202	Xz888	0.74	0.43	0.62	0.55	0.52	0.47	0.53	No	No	
…	…	…	…	…	…	…	…	…	…	…	
222	Zm1279	0.34	0.83	0.54	0.81	0.74	0.94	0.78	Yes	Yes	
223	Zms61	0.56	0.55	0.77	0.29	0.46	0.26	0.30	No	No	
224	Zms71	0.47	0.83	0.46	0.51	0.48	0.62	0.64	No	Yes	
225	Zz14	0.47	0.41	0.38	0.04	0.77	0.11	0.25	No	No	
	Mean^#^	29.92	30.13	5.12	98.65	92.87					
	Maximum^#^	32.70	33.60	5.80	111.78	97.52					
	Minimum^#^	26.85	26.00	4.50	84.50	87.00					

^*^The full genotype by trait table is graphically displayed in Fig. 1. The traits are max-scaled with 1 being the highest and 0 the lowest. The selection index (SI) was calculated as a linear combination of the traits with weights of −0.17, 0.30, −0.19, 0.93 and 0.14 for fiber length, fiber strength, micronaire, yield and pre-frost yield rate (PFR), respectively. The adjusted selection index (ASI) was calculated with weights of 0.30, 0.40, −0.40, 0.60 and 0.10 for traits in the same sequence above. ^**^ “H”: high level and “L”: low level for the relevant trait in independent culling. ^#^Statistics derived from the real value.

First, the GT-biplot showed the associations between the traits across the 225 genotypes: (1) a high and positive correlation (an acute angle) between SI and lint cotton yield (yield), (2) positive correlations (acute angles) between length and strength, between the pre-frost yield rate (PFR) and micronaire, and between PFR and yield, (3) negative correlations (an obtuse angle) between yield and fiber quality traits (length, strength and micronaire), and (4) negative correlations (obtuse angles) between micronaire and fiber length and fiber strength.

Second, the proposed ASI was better than the SI in integrating quality traits. The ASI was located intermediately between the yield and main fiber quality traits (length and strength); it was highly and positively correlated with fiber length, strength, lint yield, and the SI, but negatively correlated with micronaire. These associations were highly consistent with the numerical Pearson correlation coefficients presented in Table 2. Although the positive association between the SI and ASI was statistically highly significant, the SI was highly related positively with yield but negatively with fiber length and strength. From the viewpoint of cotton breeding, negative associations between yield and fiber length and fiber strength are undesirable, unlike the negative correlations between micronaire and other target traits (except for PFR). Registration decisions based on the SI effectively favor genotypes with high lint yield but low fiber quality. Decisions based on the ASI would induce a better trade-off between yield and quality.

**Table 2:** Pearson correlation coefficients for five cotton traits and the selection index. *^,^ **Significant correlation at the 0.05 and 0.01 probability levels.

Trait	Length	Strength	Micronaire	Yield	PFR	SI
Upper half mean fiber length (Length)						
Fiber strength (Strength)	0.424^**^					
Micronaire value (Micronaire)	−0.443^**^	0.118				
Lint yield relative to check (Yield)	−0.195^**^	−0.341^**^	−0.059			
Pre-frost yield rate (PFR)	−0.375^**^	−0.229^**^	0.100	0.208^**^		
Selection index (SI)	−0.183^**^	−0.155^*^	−0.102	0.965^**^	0.301**	
Adjusted selection index (ASI)	0.461^**^	0.256^**^	−0.502^**^	0.653^**^	0.032	0.747^**^

Third, the GT-biplot clearly showed the trait profiles of the genotypes to help in registration decisions or in the identification of valuable genotypes for breeding programs. In the GT-biplot, genotypes placed farther away from the biplot origin had more extreme trait profiles as opposed to the more balanced profiles of genotypes located near the biplot origin. For example, it showed that genotypes #126 and #123 had an extremely long fiber length; genotypes #190 and #78 had high micronaire but low fiber length; genotypes #147 and #157 had both extremely high SI and ASI values; and genotypes #116 and #158 had extremely high yield and SI values, but close to or lower than average levels for the other traits (except for PFR). Genotypes with balanced trait profiles, near the biplot origins, were eligible for registration while genotypes with extreme trait profiles may or may not be qualified as a new cultivar, but may be valuable as breeding parents.

### Genotype evaluation based on the selection index vs. the adjusted selection index

**Figure 2:**
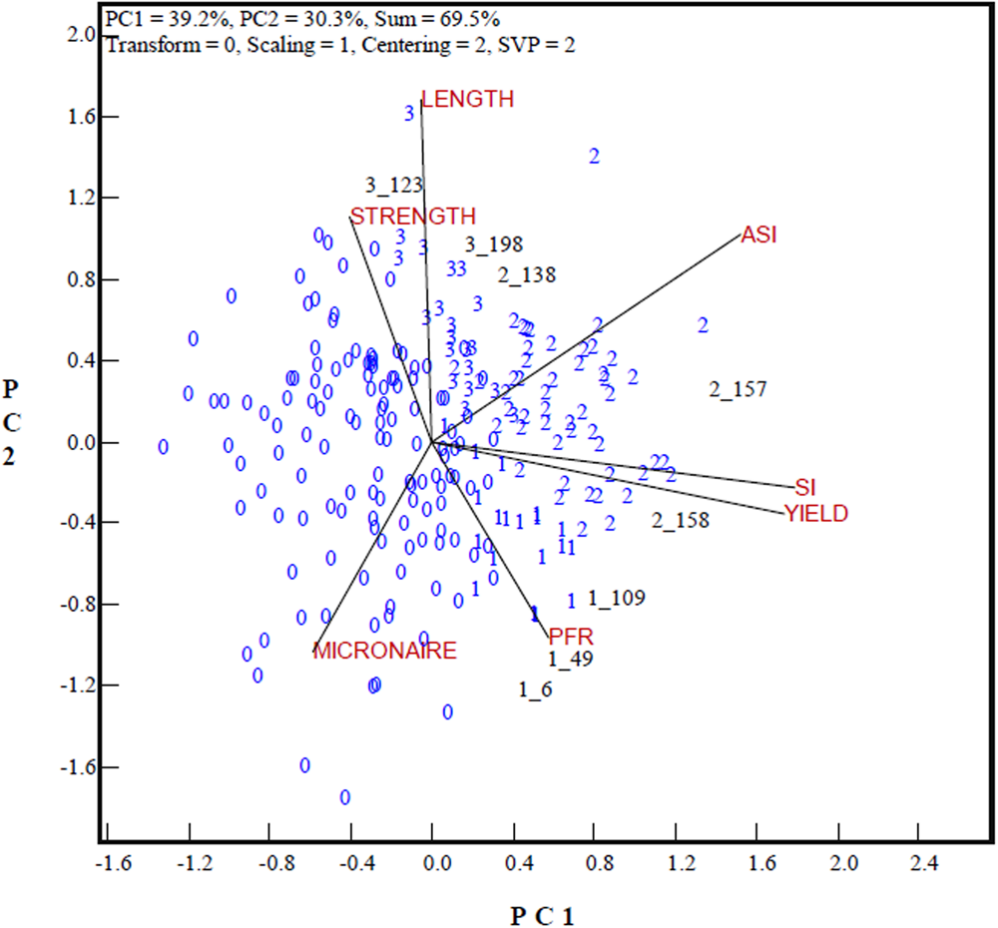
GT-biplot to show the trait associations across genotypes and genotypes selected based on the selection index (SI) vs. the adjusted selection index (ASI). “1” represents genotypes selected for a high SI but denied for the ASI; “2” represents genotypes selected for both a high SI and ASI; “3” represents genotypes selected for a high ASI but denied for the SI; “0” presents genotypes that were denied for a low SI and ASI. “1_6” represents genotype #6 selected for a high SI (indicated by 1) while “3_123” represents genotype #123 selected for both high SI and ASI (indicated by 3). Refer to Fig. 1 for trait abbreviations.

In China, the selection index, a simple linear combination of target traits^6^, is the primary factor in deciding whether a genotype is eligible for registration. Table 1 presents the values of a sample of genotypes for individual target traits and for the SI and ASI. In the past decade, 75 out of 225 candidate genotypes were found to be eligible for registration based on the values of their SI, with high projections on the SI axis, which was highly correlated with the lint yield vector. For projection on the ASI axis, 75 genotypes would have been eligible too, but only partially similar (Fig. 2). Fifty-three genotypes would have been selected according to both their SI and ASI values (labeled with “2”, e.g., 2_138, 2_157 and 2_158, the number after the underscore being the genotype code). These genotypes were mostly positioned between the yield trait and fiber quality trait vectors, indicating good combinations of lint yield and fiber quality. Twenty-two genotypes would have been selected for their SI but not for their ASI (labeled with “1”, e.g., 1_6, 1_49 and 1_109). These genotypes tended to have high levels of lint yield but poor fiber quality. On the other hand, another twenty-two genotypes would have been selected for their ASI but not for their SI (labeled with “3”, e.g., 3_123 and 3_198). These genotypes tended to have high levels of fiber length and fiber strength but low levels of lint yield. Genotypes (labeled with “0”) were denied for both the SI and the ASI.

### Independent culling based on the cotton registration standard in China

The need for correction by independent culling was slightly less when the ASI was applied compared to the SI (Table 1, genotypes discarded at the index selection stage were not considered at the independent culling stage). In China, application of the selection index is complemented by independent culling to make the final decision so as to prevent genotypes with serious defects from being registered as cultivars, given that registered cotton cultivars must have fiber length ≥25 mm, fiber strength ≥28 cN/tax, micronaire between 3.4 and 5.5, and lint yield ≥95% of check cultivars. Eight genotypes selected for their high SI values were discarded, six for poor fiber strength (i.e. genotypes #1, #109, #48, #141, #49 and #6) and two for an undesirable micronaire value (#114 and #135). Only four genotypes (#126, #148, #123 and #81) selected by the ASI were denied for low yield. There was only one genotype (#158) selected by both the SI and the ASI but culled for low fiber strength.

**Figure 3:**
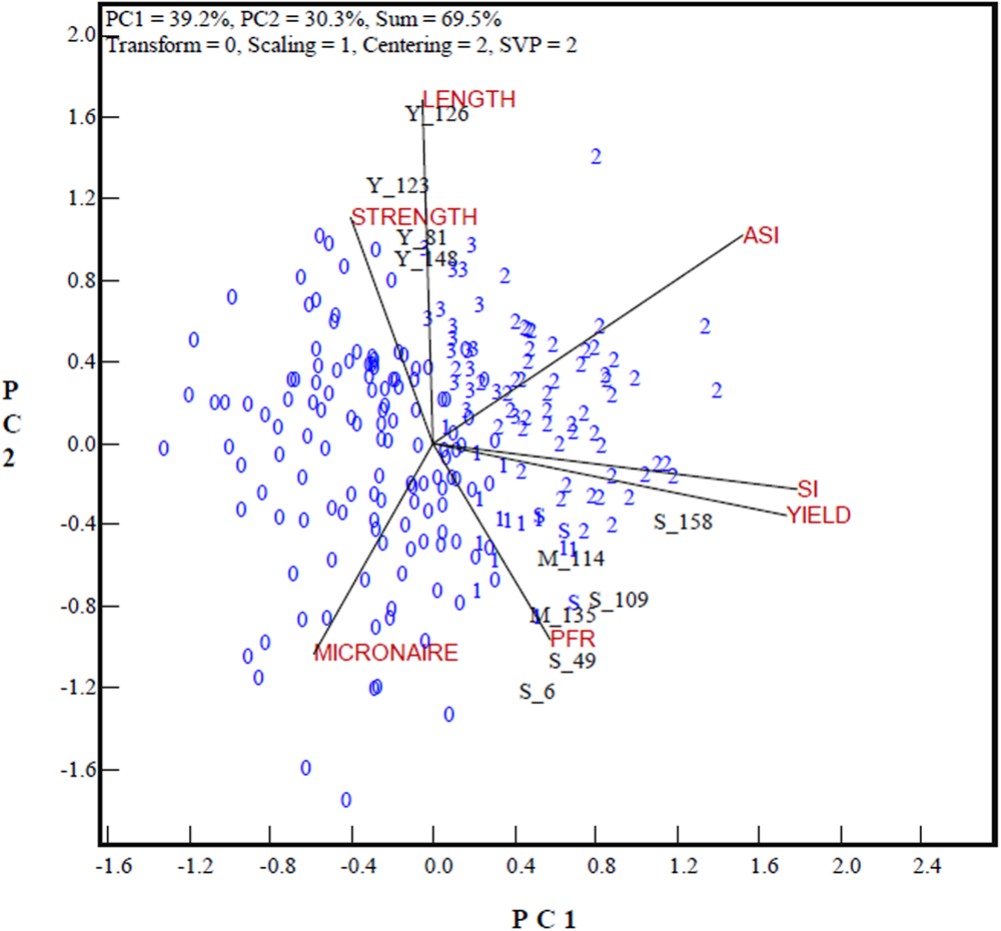
GT-biplot to show the genotypes selected based on SI vs. ASI and the independent culling result. Genotypes beginning with an upper case letter “Y”, “S” and “M” represent genotypes qualified for the SI or ASI but denied in independent culling for a poor performance in yield, fiber strength and micronaire, respectively. “S_6” represents genotype #6, which qualified for the selection index but was culled for poor fiber strength while “Y_123” represents genotype 123 culled for poor yield. Refer to Fig. 1 for trait abbreviations and Fig. 2 for the genotype group code.

By examining the trait profiles of independently culled genotypes as displayed in Fig. 3, recourse to the ASI instead of the SI could lead to a better outcome for dealing with the antagonism between yield and quality traits. It can be seen that most genotypes discarded for fiber quality traits had high projections on the lint yield axis (e.g. S_6, S_49, S_109, M_114 and M_135), while genotypes denied for low yield appeared in the sector of fiber length and strength (Y_126, Y_123, Y_81 and Y_148). The genotypes culled for low yield in Fig. 3 were all labeled as “3” in Fig. 2, and the genotypes culled for fiber quality traits were labeled as “1” in Fig. 2, implying that genotypes with extreme trait profiles selected by the SI and/or ASI were effectively eliminated by implementing independent culling. As a result, 52 out of 53 genotypes selected by both the SI and ASI successfully passed independent culling and were qualified for registration. Application of the ASI would have discarded for registration 14 of the 75 genotypes supported by the SI and would have supported 18 other genotypes.

### Genotype grouping and trait profile comparison

As shown in Fig. 3, the candidate genotypes were clustered in four groups after independent culling, (1) the discarded genotypes denied by both the SI and ASI in the bottom left-hand sector of the biplot (labeled “0” in Fig. 3) can be called “Group 0”; (2) “Group 1” included genotypes that passed the SI and independent culling but were denied by the ASI, which were placed within or around the sector between the yield and PFR vectors (labeled “1” in Fig. 3); (3) “Group 2” covered genotypes selected by both the SI and ASI, and which also passed independent culling (labeled “2” in Fig. 3); and (4) “Group 3” represented genotypes selected by the ASI and also passed the independent culling, located within the sector between the fiber length and ASI vectors (labeled “3” in Fig. 3).

In order to clarify the specific characteristics of cotton genotypes in different groups, a one-way analysis of variance (ANOVA), followed by a LSD (Least Significant Difference) test were performed to compare the groups for key traits. Table 3 shows that the greatest differences between the genotype groups were for lint yield. The yield improvement of genotypes in Group 2 was about 10% over Group 0, while genotypes in Group 1 and Group 3 increased about 8 and 3% respectively. The fiber length of Group 3 was significantly higher than the other groups, while Group 1 was significantly lower than Group 0 in fiber length. The fiber strength of Group 3 also ranked first and was significantly higher than the other groups, without any significant differences between the other groups. The micronaire of genotypes in Group 2 and 3 was also significantly improved over Group 1 and Group 0. The difference in PFR between groups was not statistically significant, implying that selection gain for this trait had little relevance to cultivar registration.

**Table 3:** Comparison of characteristics for different groups of cotton lines in Chinese cotton regional trials in the Yangtze River Valley from 2006 to 2015.

Character	Group 0 (mean ± *SD*)	Group 1 (mean ± *SD*)	Group 2 (mean ± *SD*)	Group 3 (mean ± *SD*)
Fiber length /mm	29.853 ± 0.85 b	29.160 ± 0.53 c	30.093 ± 0.61 b	30.610 ± 0.62 a
Fiber strength/(cN/tex)	30.128 ± 1.53 b	29.901 ± 1.56 b	30.099 ± 1.15 b	31.120 ± 0.97 a
Micronaire	5.187 ± 0.19 a	5.236 ± 0.14 a	5.000 ± 0.15 b	4.988 ± 0.13 b
Pre-frost yield rate/%	92.672 ± 2.21 a	93.777 ± 1.85 a	93.140 ± 1.45 a	92.408 ± 1.41 a
Lint yield relative to check /%	95.181 ± 4.41 c	103.294 ± 2.40 a	105.084 ± 3.04 a	98.268 ± 1.52 b

Groups 1,2,3 and 0 represent genotypes selected for a high SI but denied for the ASI, selected for both a high SI and ASI, selected for a high ASI but denied for SI and that were denied for both the SI and ASI, respectively. The values followed by different lowercase or uppercase letters in the same row are significantly different at the 5 or 1% probability level.

The current cotton cultivar SI in the registration scheme in China has been highly effective in improving lint yield, but has not been effective in improving fiber quality traits. On the other hand, the ASI effectively promoted fiber quality by increasing the weight of fiber length, strength and micronaire but with a penalty for yield of 5% compared to Group 1, but still significantly higher than Group 0. In short, the antagonism between yield and quality in genotypes of Group 2 (selected by both the SI and ASI) was quite effectively handled, with the highest yield and preferable fiber quality traits. Group 1 (selected by the SI only) had relatively high yield but its fiber quality was even poorer than the discarded genotypes. Group 3 performed outstandingly for fiber quality, with lower yield, but still significantly higher than for the discarded ones. Compared to Group 2, it should be noted that Group 1 had no advantages in terms of target traits, but Group 3 stood out for fiber quality aspects and could act as an important supplement to Group 2, in order to enrich the registered cultivars with those displaying high quality traits.

## Discussion

Undesirable associations of breeding target traits are one of the most intractable challenges facing crop breeders^12^. Selection for one trait may result in changes in other important objective traits. A realistic strategy is to combine multiple traits with acceptable levels within a single cultivar. Upland cotton (*Gossypium hirsutum* L.) is a typical case of negative associations between yield and quality traits in crop breeding populations, hampering the combination of high yield and premium fiber quality in a single variety^14,15^. Past efforts to break the negative association between lint yield and fiber quality in cotton breeding have rarely been successful^15^.

With a view to managing selection criteria to breed new varieties for both yield and fiber quality, our study contributes to a better understanding of the associations between yield and fiber traits that former studies have addressed, but incompletely. Zeng and Meredith (2009)^15^ summarized the results of a number of studies on associations between lint yield and fiber quality^16–21^, and concluded that lint yield was negatively correlated with fiber strength while being positively correlated with micronaire. However, among the studies, the relationship between yield and fiber length was somewhat inconsistent compared to that between yield and strength. With regard to work based on genotypes tested in China, Xu and Li (2014) reported a negative correlation for yield vs. fiber length, and a positive relationship for yield vs. pre-frost yield rate (PFR, as an indicator of earliness)^22^. Li and Yuan (2013) concluded that cotton earliness had a negative genetic correlation with fiber quality^4^.

Our study shows a clearer relationship between yield and quality traits. In earlier studies, the associations between yield, earliness and fiber quality were rarely reported simultaneously or demonstrated with the intuitive graphics mode. In our study, the favorable or unfavorable interrelationships between yield, earliness and fiber quality properties were visually displayed in a genotype-by-trait biplot (Fig. 1), which confirmed the positive correlation of yield vs. fiber length, yield vs. earliness, fiber length vs. strength, and the negative associations between yield traits (yield and PFR) vs. fiber quality traits (fiber length and strength). Figure 1 also shows the included angle between the yield and fiber length vectors; it was an obtuse angle but close to the right angle, hence helped to explain why the results were inconsistent for the relationship between yield and fiber length in the abovementioned different research reports. At the same time, our study revealed that micronaire was negatively related to yield, fiber length and fiber strength, which was a clearer relationship than that concluded by Zeng and Meredith (2009)^15^ when analyzing a particular introgressed population of cotton, by observing that fiber strength was negatively correlated with fineness but positively correlated with the maturity ratio, while micronaire is a trait combining fineness and maturity. Nevertheless, our result has a practical implication by indicating that micronaire is probably not an obstacle to the improvement of both the yield and fiber quality considered (length and strength), hence alleviating the antagonism between yield and fiber quality traits and making the objective of balanced selection between yield and fiber quality traits realistic.

In earlier studies, recourse to the SI was considered to handle the breeding of genotypes with antagonist traits, but they only took quality traits partially into account with weights arbitrarily attributed to the integrated traits. The selection index, as a linear combination of cultivar traits with a weight assigned to each trait, was originally intended for use in breeding programs and also widely adopted by official crop committees in cultivar registration. The weights are subjective and can vary with breeding and/or registration programs, historical periods, countries, regions, and mega-environments. In the cotton variety registration scheme in Louisiana, Blanche and Myers (2006) proposed a selection index composed of a weight of 0.60 for cotton lint yield and 0.40 for fiber length^5^. Their index has the merits of being simple but some important traits such as fiber strength and micronaire were excluded. In cotton cultivar evaluation trials in Spain, Baxevanos *et al*. (2008) adopted a more complete selection index where SI = 0.6 (yield) + 0.1 (lint percentage + fiber length + fiber strength) + 0.05 (uniformity + elongation)^23^, in which micronaire remained ignored. Besides, the lint percentage (lint %) is well known to be highly correlated with lint yield, so it can be argued that there was some redundancy in the formula. Xu *et al*. (2014) proposed a selection index where SI = 0.40 (yield) + 0.13 (fiber strength) + 0.09 (fiber length + micronaire + *Verticillium*) + 0.11 (*Fusarium*) + 0.10 (PFR)^22^, which covered the most important cotton breeding objectives, but the inclusion of resistance/tolerance to cotton diseases in an index is debatable because its measurement is only artificially quantitative.

The case addressed in China shows that the effectiveness of the SI, because it is frequently arbitrarily determined, can be improved by a more objective integration of selection traits through a better understanding of trait associations. In the national cotton registration standards currently implemented in China^6^, the selection index can be presented as a linear combination: SI = −0.17 (fiber length) + 0.30 (fiber strength) −0.19 (micronaire) + 0.93 (yield) + 0.14 (PFR). In this index, negative weights were given to traits known for their negative correlations with yield (fiber length and micronaire) implying much more emphasis on yield. Eventually, registered cultivars based on this selection index had significantly higher yield but lower fiber quality (Table 3). This clearly indicates a need to increase the weight of fiber quality traits such as in the adjusted selection index (ASI) with weights of 0.30, 0.40, −0.40, 0.60 and 0.10 for fiber length, strength, micronaire, yield and PFR respectively. Intentionally, the weight sum for fiber length and strength on the one hand was preset to equal that of yield and PFR on the other hand with a view to a balanced selection index for yield and quality. For each quality or yield trait, the values of the weights in our ASI resulted from the GT-biplot analysis implemented over 10 years so that yield and fiber quality traits were equally handled (see the ASI vector Fig. 1) and much less yield-biased than with the application of the SI.

In a crop registration system or cultivar breeding programs, the application of a selection index is always accompanied by independent culling^12^. Our study showed that even though the selection index can be improved through better management of trait associations, independent culling still remains desirable. Index selection is aimed at super performance in many target traits on average, while the function of independent culling is to cut off certain genotypes with an extremely poor performance for some individual traits. In our study, although a large majority of genotypes supported by their ASI values successfully passed independent culling (52 out of 75), one third did not pass and a number of genotypes not supported by their ASI values were eventually adopted for registration. Whatever the selection index is (e.g. SI, ASI or those in other forms), it tends to support or to discard genotypes with extreme values for the traits considered. So, the implementation of independent culling is desirable to prevent the extreme candidates from being selected. Nevertheless, this observation does not question the overall value of using a selection index. In our study, a multiple trait comparison of the groups of genotypes selected by only the ASI (Group 3), by only the SI (Group 1), by both the ASI and SI (Group 2), and by the discarded genotypes (Group 0) confirmed the distinct effectiveness of the ASI in improving fiber quality and countering the long lived bias in favor of yield.

Lastly, our study showed that the GT-biplot is suitable for evaluating genotypes based on multiple traits and in discovering associations between traits, index selection and independent culling^13^. Our study further demonstrated that the GT-biplot is a powerful tool for exploring multi-trait data^11^. It visually displays the genotype-by-trait (GT) table and allows visualization of the associations between traits. The cosine of the angle between the vectors of any two traits approximates the Pearson correlation between them^13^. For example, Fig. 1 shows that the SI was highly significantly correlated with yield, and negatively correlated with the fiber quality traits, but the ASI was placed between the yield and fiber quality traits. The application of a GT-biplot can help measure the effects of a selection strategy, notably the undesired effects, hence identify ways of correcting to improve breeding programmes.

## Methods

### Ethics statement

We declare that no specific permits were required for the described locations/activities. We also confirm that the field studies did not involve any endangered or protected species.

### The selection index in the cotton cultivar registration standards in China

**A c**rop cultivar registration system is an important guarantee for the use of superior cultivars in crop production and ensures the security of agricultural production^24^. The national-level cotton variety registration scheme was launched in 1990 based on the comprehensive performance of multiple traits in cotton regional trials. However, the evaluation was somewhat subjective and there was no unified criterion for cotton variety evaluation and selection until the issue of the *Standards of Registration for Cotton Varieties* by the Chinese Ministry of Agriculture in 2007^6^. The standard presets a basic index, a basic score, or a top score for each target trait as well as rules for adding or subtracting scores based on the trait value. As the most important indicator for the final decision-making, the registration score is determined by the summation of scores from each target trait. The role of the registration score is equivalent to a selection index, which is not a linear combination of target traits. The calculation of the registration score is complicated and laborious, but it can be renamed as a “selection index” and simplified by a regression analysis as a linear combination of the target traits with weights of −0.17, 0.30, −0.19, 0.93 and 0.14 for fiber length, fiber strength, micronaire, yield and PFR, respectively.

### National cotton regional trials in the Yangtze River Valley

he national regional cotton variety trials over the 2006–2015 period in the Yangtze River Valley (YRV) included 18 test locations covering eight provinces, namely, Sichuan, Hunan, Hubei, Henan, Jiangxi, Anhui, Jiangsu and Zhejiang^25^. The trials were sponsored by the Chinese Ministry of Agriculture and coordinated by Jiangsu Academy of Agriculture Sciences with the objective of recommending varieties suitable for national registration and commercial release on a trans-provincial scale in YRV. The entries varied from year to year. New entries were added each year and only entries with a good overall performance in the first year were advanced to the second year test. The candidate varieties tested amounted to 225 over the period. All the field trials in each test location were laid out in a randomized complete block design with three replications. The plot size was 20 m^2^ with 3 to 6 rows. The space between and within rows varied from 80 to 110 cm and from 30 to 40 cm, respectively, depending on the year and location. Raising seedlings in a nutritious block followed by transplanting was the major planting procedure. Pre-transplanting fertilizers were applied as follows: 165 kg N/ha, 79 kg P/ha, and 113 kg K/ha. Additional fertilizers were applied during the blooming stage at a rate of 110 kg N/ha and 49 kg K/ha. Other cultivation practices were similar to local practices. During cotton growth and development, 20 plants were sequentially labelled in each plot for the recording of agronomic traits. Seed cotton was harvested several times by hand up to November 20, the unified deadline for the total cotton yield estimation; the harvest percentage before November 10 was defined as the pre-frost yield rate (PFR), which was used as the main indicator of variety earliness. During the cotton boll opening period, 50 normally opened bolls of each variety were collected as lint samples for fiber quality testing. Several fiber quality traits were measured by the Cotton Quality Supervision and Testing Centre of the Chinese Ministry of Agriculture using a high-volume instrument (HVI) testing system but only three fiber traits, i.e. fiber length, fiber strength and micronaire, were adopted for national cotton variety registration.

### Data analysis

For the dataset of 225 candidate genotypes from regional trials in the Yangtze River Valley, five target quantitative traits were measured/evaluated: fiber length, fiber strength, micronaire, yield increasing rate and pre-frost yield rate (PFR). Yield was reported as lint yield relative to the common check cultivar “Ezamian 10”. The selection index acted as the most important criterion in deciding whether an entry was supported for registration. Independent culling was applied to ensure that the cultivars met minimum requirements for key traits and other considerations for a cultivar. Specifically, a candidate variety was culled if its fiber length was less than 25 mm, or fiber strength was less than 28 cN/tex, or micronaire was smaller than 3.4 or larger than 5.5, or lint yield was less than 95% of the check. A GT-biplot analysis was conducted to visualize the relationships existing between the five quantitative objective traits, their SI and/or ASI, as well as the trait profiles of the registered/selected genotypes. The GT-biplot was based on the following formula^11^:

1 $$\frac{{{\rm T}}_{\iota j}-{\bar{{\rm T}}}_{j}}{{s}_{j}}={\lambda }_{1}{\zeta }_{{i1}}{\tau }_{{1}j}+{\lambda }_{{2}}{\zeta }_{i{2}}{\tau }_{{2}j}+{\varepsilon }_{ij}$$

where ${{\rm T}}_{\iota j}$ is the value of genotype *i* for trait *j* in the GT-table, ${\bar{{\rm T}}}_{j}$ is the average value of trait j over all genotypes, ${s}_{j}$ is the standard deviation of trait *j* among the genotype averages; ${\zeta }_{{i1}}$ and ${\zeta }_{i{2}}$ are the PC1 and PC2 scores, respectively, for genotype *i*; ${\tau }_{{1}j}$ and ${\tau }_{{2}j}$ are the PC1 and PC2 scores, respectively for trait *j*, and ${\varepsilon }_{ij}$ is the residual of the model associated with genotype *i* in trait *j*. A GT-biplot is constructed by plotting the first principal component (PC1) scores of the genotypes and the traits against their corresponding scores for the second principal component (PC2) resulting from singular-value decomposition (SVD) of trait-standardized data. In the “Relationship between testers” view of the GT-biplot, the correlation coefficient between any two traits is approximated by the cosine of the angle between their vectors^26,27^. An ANOVA and multiple comparisons were performed for key traits to compare different groups of genotypes separated by the SI and ASI.

### Data availability statement

All relevant data are within the paper and its Supporting Information files.
